# Classic Kaposi Sarcoma: A Comprehensive Case Report on Multisite Involvements and Therapeutic Strategies

**DOI:** 10.7759/cureus.52553

**Published:** 2024-01-19

**Authors:** Leo Wan, Audrey Yan, Casey O'Doherty, Xiner Jiang, Rosemarie Hardin

**Affiliations:** 1 Medicine, West Virginia School of Osteopathic Medicine, Lewisburg, USA; 2 Biochemistry, Stony Brook University, Stony Brook, USA; 3 Plastic Surgery, Wheeling Hospital, Wheeling, USA

**Keywords:** hhv-8, classic kaposi sarcoma, kaposi sarcoma treatment, clinical dermatology, kaposi sarcoma hiv negative

## Abstract

Classic Kaposi sarcoma (CKS), a variant of Kaposi sarcoma (KS), predominantly affects elderly men of Mediterranean and Ashkenazi descent. It is primarily seen in immunocompetent patients, often as cutaneous manifestations in the lower extremities. Treatment of CKS ranges from radiation therapy, chemotherapeutic agents, surgical excision, cryosurgery, and immunotherapy, and the treatment selection is contingent on disease-specific manifestations. This study presents the case of an 83-year-old immunocompetent male of Mediterranean descent, diagnosed with CKS five years ago, exhibiting an onset of painful violaceous papulonodular lesions on the right medial plantar surface and painless papulonodular lesions on the right upper arm and medial thigh. The case highlights the intricacies of CKS diagnosis and management, shedding light on the diverse treatments targeted for lesions across various anatomical locations.

## Introduction

Kaposi sarcoma (KS) is a rare vascular tumor first identified by Dr. Moritz Kaposi in 1872 from five cases of "idiopathic, multiple pigment sarcoma of the skin" [1-3]. Originating from the lining of lymph or blood vessel cells, KS is categorized into four subtypes: classic, endemic, iatrogenic, and acquired immune deficiency syndrome (AIDS)-associated [1-3]. Common manifestations include cutaneous lesions appearing as purplish or reddish-brown macules, papules, or nodules potentially spreading to multiple visceral organs [1]. Although clinical presentations may appear similar, the variant and disease progression depend on the host's immune status, ethnicity, and age. Frequently associated with acquired immunodeficiency syndrome (AIDS) and human herpesvirus-8 (HHV-8), KS can also affect immunocompetent individuals [1].

Unlike other variants linked to immunosuppression, classic Kaposi sarcoma (CKS) predominantly occurs in immunocompetent patients [2]. Lesions typically present in lower extremities as asymptomatic bruises, progressing into plain maculae or patches and elevated plaques. Lesions are occasionally preceded by or associated with non-pitting edema. Levi initially developed the staging of CKS: limited skin involvement in stage I, disseminated skin involvement in stage II, gastrointestinal tract and lymph nodes in stage III, and disseminated skin lesions spreading to visceral organs and lymphatic or pulmonary lesions in stage IV [2].

The rarity of CKS makes accurate diagnosis difficult, potentially misdiagnosing it as a common skin condition such as arterial insufficiency or venous stasis wounds. Treatment options are available for KS, and the choice(s) depends on clinical form, stage, size, and location [1]. Treatment modalities include radiation, surgical excision, cryosurgery, immunotherapy (such as interferon alfa-2b), and chemotherapeutic agents such as pomalidomide [2-5].

## Case presentation

**Figure 1:**
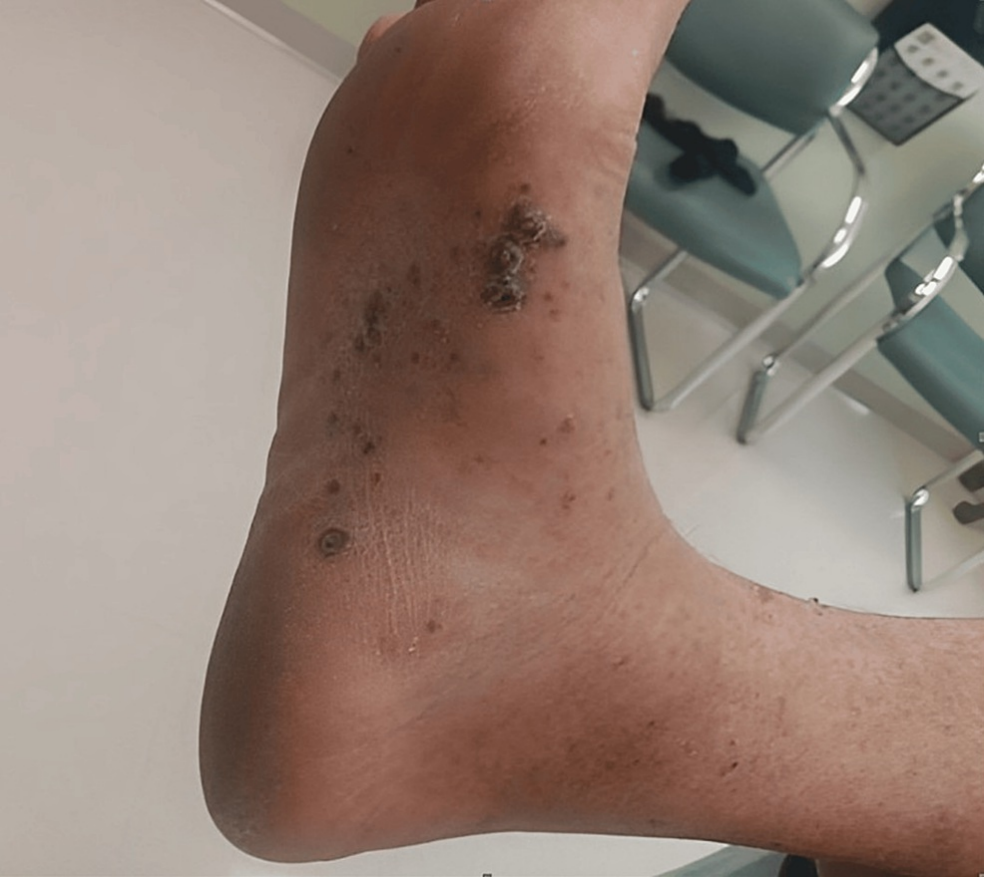
Violaceous papulonodular lesions on the right medial plantar surface.

**Figure 2:**
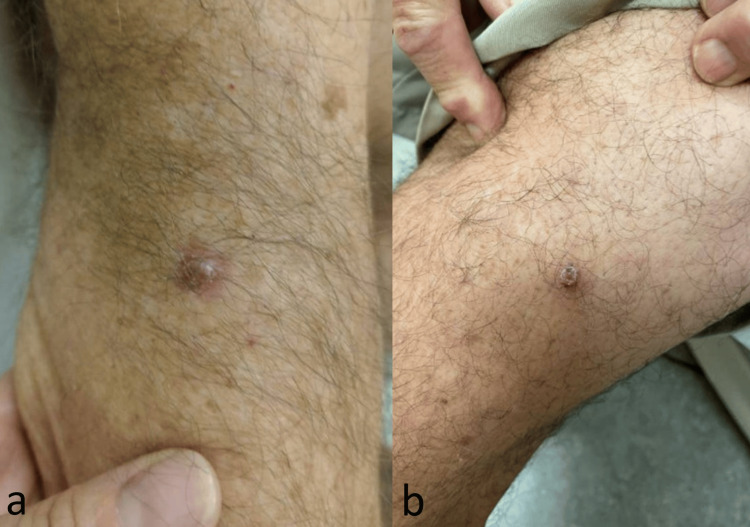
(a) Solitary papulonodular lesion on the right upper arm. (b) Solitary papulonodular lesion on the right medial thigh.

An 83-year-old male patient of Mediterranean descent presented to our clinic with a history of hypertension, hyperlipidemia, and a confirmed CKS diagnosis. The visit was prompted by the recent onset of painful violaceous papulonodular lesions on the right medial plantar surface while ambulating (Figure 1) and a painless papulonodular lesion on the right upper arm and right medial thigh (Figure 2a, 2b).

 

**Figure 3:**
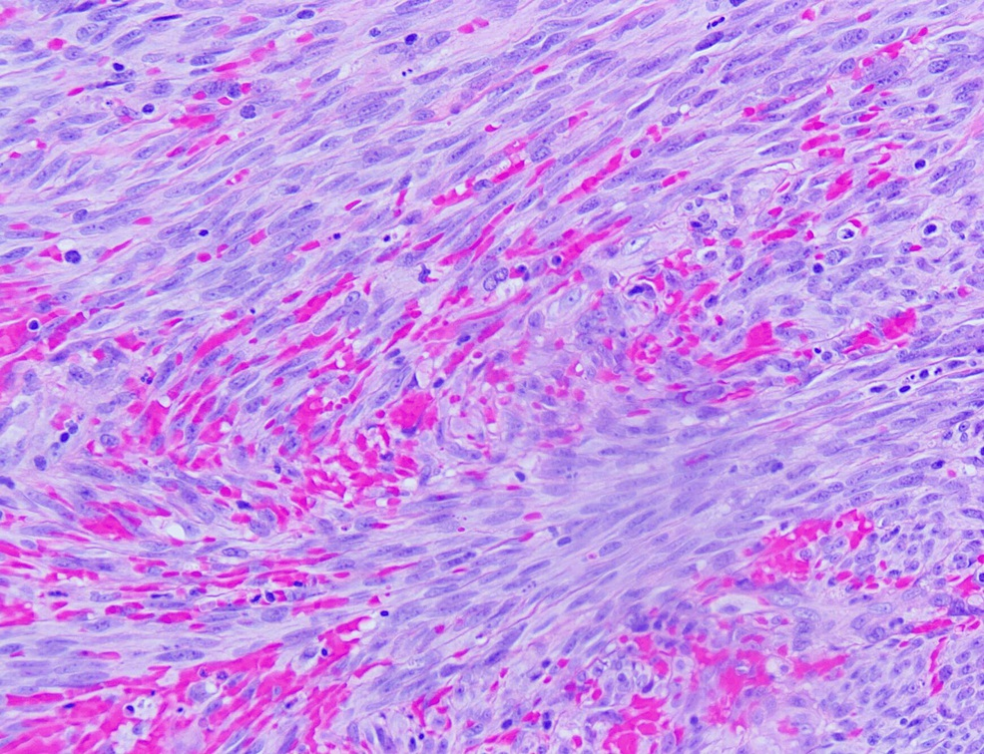
Shaved biopsy at the right medial plantar surface reveals spindle cell proliferation with mitosis, heightened vascularity, and red blood cell extravasation.

**Figure 4:**
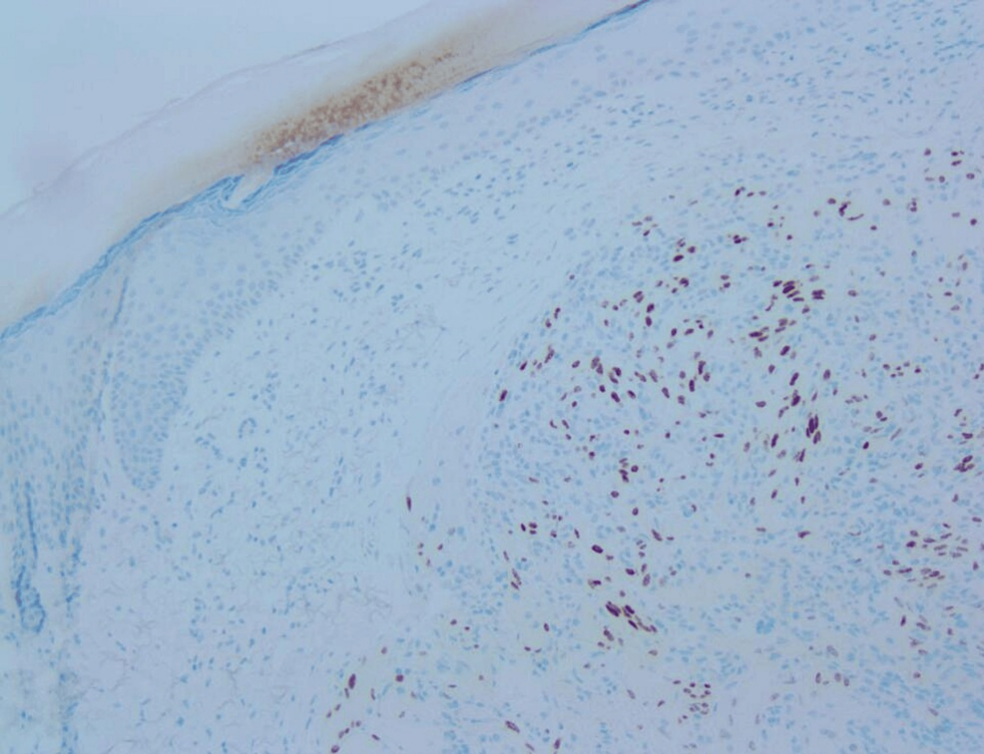
HHV-8 stain performed confirming the diagnosis of CKS. HHV-8: human herpesvirus-8; CKS: classic Kaposi sarcoma

The patient's five-year history of CKA-associated cutaneous manifestations initiated with the first lesion on the right ear. CKS diagnosis was histopathologically confirmed by a biopsy and a positive HHV-8 stain, subsequently managed by surgical excision (Figure 3 and Figure 4). Two years after the initial diagnosis, additional lesions manifested on the fourth left finger, instep of the right foot, left lateral ankle, left upper arm, and right medial foot and were therapeutically addressed by radiation therapy.

Standard complete blood count yielded no noteworthy findings. The human immunodeficiency virus (HIV) serology returned negative results. Shaved biopsies revealed spindle cell proliferation with mitosis, heightened vascularity, and red blood cell extravasation (Figure 3).

Due to the lesion's location on the right medial plantar surface and the discomfort during walking, the patient opted against surgical excision. Consequently, a referral to the radiation oncology department at a tertiary care center was initiated. The patient underwent treatment with a cycle of pomalidomide, an antineoplastic medication.

## Discussion

CKS is an uncommon malignancy of vascular endothelial origin, characterized by bruises on the lower extremities evolving into dark papules and plaques. While the disease progression is typically slow, aggressive forms can result in severe complications, including rare occurrences of metastasis to other organ systems. Moreover, a notable association between CKS and HHV-8 has been suggested [2]. The escalation in anti-HHV-8 antibodies has been correlated with increased susceptibility to KS development, revealing a potential role in the pathogenesis [6]. Polymerase chain reaction (PCR) analysis conducted on 41 KS patients revealed that 40 were positive for HHV-8, conveying a significant correlation between the virus and KS [6].

Furthermore, rodent fibroblast transformation assays have demonstrated plausible HHV-8 oncogenic traits in at least four genes associated with CKS malignancy [7]. The findings have contributed to two potential models for KS pathogenesis from HHV-8: (1) a classical model akin to other virus-induced models and (2) a model induced by chronic inflammation from HHV-8 cytokine release [7]. These insights offer valuable perspectives for understanding the complex interplay between HHV-8 and CKS development. 

The therapeutic approach for KS exhibits variability contingent upon the clinical form and stage in addition to lesion location and size. Available options include, but are not limited to, surgical options, local or extended field radiotherapy, and chemotherapy [3]. Excisional surgery is a therapeutic approach for diagnosing and managing large, ulcerating bleeding nodules in classic nodular CKS patients [4]. However, its efficacy may be compromised, especially in elderly individuals with CKS, due to the prevalence of lower limb lesions. Initial excisional surgery on lower limbs often necessitates additional procedures, such as flaps or skin grafts, but entails increased financial and time costs. Excisional surgery may result in prolonged recovery periods and heightened infection risks. Additionally, extended surgical trauma elevates the risk of lymphatic system impairment, thereby amplifying the potential for subsequent lymphedema [4].

Radiation therapy stands out as the most efficacious treatment modality in CKS. It has been extensively utilized for patients presenting with large, multiple, and unresponsive lesions to alternative treatment approaches. A study by McGill University comprising 16 CKS patients revealed a mean age of 74 years at diagnosis among 13 male patients. Ninety-four percent of patients exhibited leg lesions, while 12.5% presented with arm lesions [3]. The frequently prescribed radiation dose was 30 Gy, administered in 15 daily fractions of 2 Gy. All lesions exhibited a positive treatment response, with an 88% complete response rate and a 12% partial response rate [3].

Pomalidomide is an oral small-molecule derivative of thalidomide with immunomodulatory properties. It targets cereblon, an E3 ubiquitin ligase, leading to downstream effects such as the modulation of tumor necrosis factor-alpha, interleukin-6, vascular epithelial growth factor, and enhancement of CD4+ and CD8+ T-cell costimulation [5].

The most common side effects of pomalidomide encompass hematological toxicities, including leukopenia, neutropenia, and lymphocytopenia. However, most episodes resolve spontaneously. In one case involving an HIV-negative KS participant, a widespread petechial rash and vasculitis led to the discontinuation of pomalidomide, but a good response was observed after a prolonged course of steroid therapy. Other toxicities include gastrointestinal symptoms such as constipation, fatigue, and acneiform rash, which responded well to hydrocortisone ointment and antihistamines [8]. Despite potential side effects, the drug has shown increased immune activity and lesion clearance in KS patients when administered at a dosage of 5 mg for 21 of 28 days, irrespective of HIV status. Pomalidomide emerges as a valuable option for individuals seeking oral therapeutics, avoiding cytotoxic chemotherapy, and avoiding the cumulative use of anthracyclines in managing refractory diseases [5].

The patient underwent various treatments tailored to their distinct clinical and anatomical presentations. The initial lesion on the right ear required surgical excision, which was imperative for histopathological confirmation of CKS diagnosis. Lesions that manifested two years after, affecting the left fourth finger, instep of the right foot, left lateral ankle, left upper arm, and right medial foot, underwent extended field radiation therapy with a dose of 30 GY administered in 15 fractions. The patient displayed good tolerance to the treatment, resulting in the desiccation of the treated areas. Subsequently, the patient adhered to a regular moisturization routine for these areas. Recent lesions affecting the right medial plantar surface, upper arm, and medial thigh were addressed with a 4-milligram pomalidomide capsule. The patient was on oral administration once daily for 21 days within a 28-day cycle. Remarkably, the skin lesions demonstrated significant improvement and approached complete resolution.

## Conclusions

While KS is traditionally linked to immunocompromised individuals, it is pertinent to acknowledge rarer variants such as CKS which predominantly occur in immunocompetent individuals. A comprehensive diagnostic approach should include HHV-8 testing in conjunction with traditional HIV serology and biopsy. When discussing treatment options with patients, the multitude of clinical manifestations of CKS must be carefully considered. Radiation therapy remains the most efficacious therapy for CKS, especially for individuals presenting with multiple or unresponsive lesions to alternative methods. Our patient initially underwent surgical excision, but full resolution was ultimately achieved after radiation therapy and pomalidomide.
